# Identification of Punicalagin as a Key Bioactive Compound Responsible for the Antimicrobial Properties of L. Peel Extract against 

**DOI:** 10.1021/acs.jafc.5c02320

**Published:** 2025-07-09

**Authors:** Amira Salim, Lewis Marquez, Kishor Jakkala, Sunmin Woo, Marco Caputo, Francesco Fancello, Pierfrancesco Deiana, Mario Santona, Maria Giovanna Molinu, Cassandra L. Quave, Severino Zara

**Affiliations:** † Department of Agricultural Sciences, University of Sassari, Sassari 07100, Italy; ‡ Molecular and Systems Pharmacology Program, Laney Graduate School, Emory University, Atlanta, Georgia 30322, United States; § Department of Microbiology and Immunology, Emory School of Medicine, Emory University, Atlanta, Georgia 30329, United States; ∥ Center for the Study of Human Health, Emory University, Atlanta, Georgia 30322, United States; ⊥ National Council of Research, Institute of Food Production Sciences, Sassari 07100, Italy; # Department of Dermatology, School of Medicine, Emory University, Atlanta, Georgia 30322, United States

**Keywords:** Staphylococcus aureus, natural products, Punica granatum, fractionation, punicalagins α and β

## Abstract

This study highlights the potential of (pomegranate) peel extract (PPE) as a natural antimicrobial alternative to synthetic chemicals. Pomegranate peels, typically considered a byproduct of fruit processing, contain bioactive compounds such as polyphenols, flavonoids, and tannins. This research explored the antimicrobial efficacy of these phytochemicals against using a bioassay-guided fractionation approach to identify key bioactive components. While evaluating antimicrobial efficacy is critical, it is equally important to ensure safety for human use. Therefore, we assessed the cytotoxicity of punicalagins α and β on human keratinocytes to determine their biocompatibility and potential for safe application in food or therapeutic settings. The antibacterial activity of PPE was then evaluated using different food models. Punicalagins α and β were identified as individual compounds, and their pure reference forms showed a minimum inhibitory concentration (MIC) of 16 μg/mL. None of the tested fractions exhibited significant cytotoxicity to human keratinocytes (HaCaTs) (IC_50_ > 128 μg/mL). A significant reduction of up to 0.8-fold in bacterial cell counts was observed after PPE incorporation into tested food models. This research underscores the importance of exploring sustainable antimicrobial solutions derived from food industry byproducts as alternatives to synthetic antibiotics.

## Graphical abstract

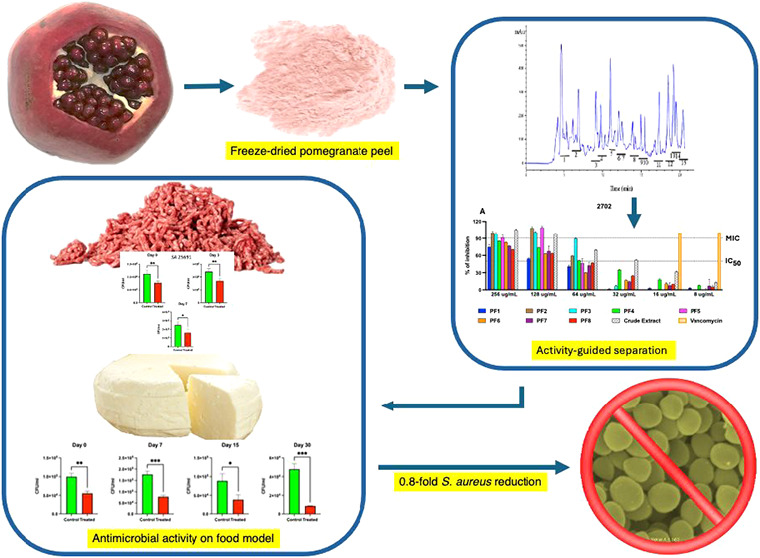

## 1 Introduction

Foodborne diseases caused by pathogens, including bacteria, viruses, fungi, prions, and parasites, found in contaminated food and water pose significant public health challenges worldwide. There are more than 200 diseases linked to contaminated food,^1^ where contaminated food results in 600 million cases of foodborne diseases and 420,000 deaths worldwide every year.^2,3^ Staphylococcal foodborne diseases are a prevalent global foodborne illness caused by the presence of bacteria in contaminated food.^4^ This pathogen poses a significant risk to consumers, leading to severe economic losses and decreased human productivity due to foodborne illnesses.^5^ Furthermore, the excessive use of antibiotics in agriculture has created favorable conditions for the exposure and dissemination of resistant strains of various pathogens.^4^ Natural compounds derived from plants have shown great promise as an alternative source of novel active molecules,^6^ whereas medicinal plants serve as important reservoirs of new molecules due to their wide range of secondary metabolites with interesting and unique pharmacophores.^7^ Pomegranate ( L. Lythraceae) peel possesses several classes of phytochemicals, including hydrolyzable tannins, phenolic acids, triterpenoids, phytosterols, lignans, and flavonoids.^8–10^ Among these phytochemicals, polyphenolic compounds have demonstrated a diverse array of bioactivities, including antioxidant, anti-inflammatory, anticancer, antidiabetic, antiatherogenic, and antifungal properties. Additionally, phytochemicals from pomegranate peels exhibit immunomodulatory activities and serve as potent inhibitors against , , , , , , and .^11–14^ Moreover, several studies propose that the ability of pomegranate extracts to inhibit quorum sensing (QS) may contribute to its effectiveness in preventing biofilm formation.

QS serves as a communication system among bacteria and facilitates interactions related to nutrients, defense against other microorganisms, virulence, and biofilm development. The QS-modulating properties of pomegranate have been linked to various polyphenols, including punicalagin and ellagic acid.^15–17^ On the other hand, a comparative study on the quorum modulatory effect of selected medicinal plants, including , found that while pomegranate peel extract exhibited violacein inhibitory activity against , the effect was less pronounced compared to other plant extracts, indicating variability in QS inhibition efficacy.^18^

PPE is a valuable byproduct in the food preservation sector due to the high content of bioactive substances found in this species.^19^ Despite significant interest in pomegranate extracts, the specific compounds responsible for their antimicrobial effects remain unclear. To date, no studies have consistently identified an identical chemical profile associated with the antimicrobial activity in PPE. These inconsistencies may result from variations in research methodologies or from differences in the phytochemical compositions of pomegranates from various varieties and geographical regions.^20^

The present study aimed to identify antimicrobial compounds from peel extracts against pathogen using bioassay-guided fractionation. Inhibition of quorum sensing was also assessed in extract fractions. The most bioactive fractions were evaluated for cytotoxicity on human skin cells. Subsequently, in-food matrix assays were performed to validate the antimicrobial efficacy of peel extracts in food models, specifically using cheese and ground meat models.

## 2 Materials and Methods

### 2.1 Chemicals

Optima LC-MS grade acetonitrile and water, containing 0.1% formic acid respectively, and methanol were purchased from Fisher Scientific (Pittsburgh, PA, USA) and Supelco (Bellefonte, PA, USA), and punicalagin (α and β mixture) was purchased from PhytoLab GmbH & Co.KG (Germany).

### 2.2 Collection of Plant Materials and Extraction

Fruits from 7 varieties of were harvested on October 20th, 2021 (Table 1). The harvest took place in the pomegranate varietal collection field of the University of Sassari’s Experimental Station “A. Milella” in Oristano, Sardinia, at coordinates 39°54′12″N, 8°37′19′′E. Herbarium specimens were collected and deposited at Emory University Herbarium (code: GEO), all specimens were digitized and are available on the SERNEC Portal (https://sernecportal.org/portal/index.php).

**Table 1:** Pomegranate Specimens’ Data

Specimen code	Family	Genus	Specific Epithet	Species Authority	Variety	Voucher Specimen Accession Number	Collection site
**2702**	Lythraceae	*Punica*	*granatum*	L.	Mollar Elche	GEO17912	Sardinia, Italy
**2703**	Lythraceae	*Punica*	*granatum*	L.	Arbara druci	GEO17919	Sardinia, Italy
**2704**	Lythraceae	*Punica*	*granatum*	L.	SS3 Antigga maddura	GEO17914	Sardinia, Italy
**2705**	Lythraceae	*Punica*	*granatum*	L.	Wonderful	GEO17918	Sardinia, Italy
**2706**	Lythraceae	*Punica*	*granatum*	L.	SS2 classica	GEO17922	Sardinia, Italy
**2707**	Lythraceae	*Punica*	*granatum*	L.	Selezione siciliana Primosole	GEO17920	Sardinia, Italy
**2708**	Lythraceae	*Punica*	*granatum*	L.	SS1 precoce	GEO17916	Sardinia, Italy

Four pomegranate fruits for each genotype were processed for extraction, where the peel and arils were manually separated and the peel was finely chopped. Subsequently, the chopped peel underwent a 72 h drying process at −55 °C using a Vacuum Electrical Defrost Dryer. The dried peel was further processed into a fine powder by using a laboratory blender (Waring Commercial Blender 7011S).

Plant material was extracted by maceration. Briefly, ground and dried peels of were macerated (ratio of 1:10 w/v) in 80% aqueous ethanol (v/v) at room temperature for 72 h under constant agitation. This process was repeated for a second time using the same plant residue to increase the extract yield. Both extraction products were filtered, then combined. The resulting alcoholic filtrate was concentrated using a rotary evaporator, shell-frozen, and lyophilized for 24 h. The resulting extracts were stored at −20 °C in a dry state until needed, at which point they were dissolved in 100% dimethyl sulfoxide (DMSO) at a stock concentration of 10 mg/mL for the assays.

### 2.3 Isolation of Bioactive Compounds

An exploratory high-performance liquid chromatography (HPLC) analysis was conducted on all of the samples. Based on the chromatographic data received from this analysis and findings from the prior study,^14^ only the most potent varieties were chosen for further investigation. extracts 2702 (, Mollar Elche variety) and 2707 ( Selezione siciliana Primosole variety) underwent bioassay-guided fractionation through reversed-phase HPLC, where method development for the crude extract fractionation was performed on preparative HPLC (prep-HPLC). All subsequent prep-HPLC were carried out using an Agilent 1260 Infinity system running OpenLab CDS ChemStation (Agilent Technologies, Santa Clara, CA, United States) equipped with a UV–vis detector, fraction collector, and Agilent XDB-C18 (21.2 mm × 250 mm, 5 μm) column with a compatible guard column. Mobile phases were 0.1% (v/v) formic acid in water (A) and 0.1% (v/v) formic acid in acetonitrile (B), at a flow rate of 21.20 mL/min, and monitored for 36 min. 2702 and 2707 crude extracts were dissolved in MeOH, and a total of 34 injections each with a 1 mL sample injection (30 mg/mL in 80:20 H_2_O/MeOH) were performed. Chromatograms were monitored at 254 and 314 nm. A custom-built open-bed fraction collector was used for fraction collection (Caputo et al., 2020). Initial conditions were 90:10 (A/B), held for 5.00 min, changing to 85:15 (A/B) for 10.00 min, changed to 67:33 (A/B) until 30.00 min, then eluted as 0:100 (A/B), from 30.01 until 36 min before returning to initial conditions to equilibrate the column. A total of 16 preparative fractions (PFs) were obtained using this method; 8 for each crude extract 2702 and 2707. Due to their activity against the tested strains, only 2707 PF5 and PF6 were chosen for further fractionation. A second round of prep-HPLC to split 2707 PF5 and PF6 into “subfractions” (SFs) was conducted using the method cited above with some change in the gradient elution. A gradient elution consisting of mobile phases (A) 0.1% formic acid in H_2_O and (B) 0.1% formic acid in acetonitrile at a flow rate of 21.20 mL/min and monitored for 35 min was 95:5 (A/B), held for 5.00 min, changing to 85:15 (A/B) for 20.00 min, then elution 0:100 (A/B), from 25.01 to 35 min before returning to initial conditions to equilibrate the column. Two runs for 2707 PF5 and three runs for 2707 PF6 were performed with a 1 mL sample injection each time (30 mg/mL in 80:20 H_2_O/MeOH). A total of 68 SFs from both 2707 PF5 and PF6 were obtained using this method.

### 2.4 LC-MS Characterization of Subfractions

For LC-MS analysis, the dried extract of 2707, its associated SFs, and the punicalagin mixture (α and β) were dissolved in a methanol/water solution (5:5, v/v). The analysis was conducted using an Agilent 1290 Infinity II UHPLC system connected to an Agilent 6545XT Quadrupole Time-of-Flight (QTOF) Mass Spectrometer (Agilent Technologies, Santa Clara, CA, USA), equipped with a dual AJS ESI ion source. Chromatographic separation was achieved by using a Zorbax Eclipse XDB-C18 column (100 × 2.1 mm, 1.8 μm) in combination with a Zorbax Eclipse XDB-C18 guard column (5 × 2.1 mm, 1.8 μm). The mobile phase consisted of water (A) and acetonitrile (B), each containing 0.1% formic acid. The column and sample organizer temperatures were maintained at 40 and 15 °C, respectively. Elution was carried out with a stepwise gradient at a steady flow rate of 0.4 mL/min under the following conditions: 5–5% B from 0.0 to 0.5 min; 5–15% B from 0.5 to 5.0 min; 15–100% B from 5.0 to 9.0 min; and 100–100% B from 9.0 to 10.5 min. The column was then returned to the initial conditions at 10.6 min, with a 1.4 min re-equilibration period, giving a total run time of 12.0 min.

Sample injections of 2.0 μL were analyzed in negative ion mode, using both profile and centroid detection. Electrospray ionization parameters were set as follows: capillary voltage at 4.0 kV, nozzle voltage at 2000 V (negative mode), fragmentor voltage at 100 V, drying gas temperature at 325 °C with a flow rate of 13 L/min, sheath gas temperature at 275 °C with a flow rate of 12 L/min, and nebulizer pressure of 35 psi. Nitrogen was used for both nebulization and drying. The Auto-MS/MS acquisition mode covered an MS range of *m*/*z* 100–1700 and an MS2 range of *m*/*z* 50–1700, operating at 7 and 5 spectra/s, respectively. A narrow isolation width (∼1.3 *m*/*z*) was employed. Collision energy was calculated based on precursor *m*/*z* and charge using two conditions: condition 1 with a slope of 3.8 and offset of 20, and condition 2 with a slope of 2.0 and offset of 6. A maximum of five precursors were selected per cycle, with an absolute threshold of 500 and a relative threshold of 0.015%. Active exclusion was applied after three spectra, and the sample was lifted after 0.1 min. Data acquisition and processing were carried out using MassHunter Workstation Acquisition B.10.00 and MassHunter Qualitative Analysis 10.0 software (Agilent Technologies).

### 2.5 Bacterial Strains

The following bacterial strains were used in this study: MRSA strain LAC (AH845), AH1677 (Quave lab, Emory University, USA), DSM 20231, and DSM 25691 (Leibniz Institute DSMZ, Germany). After streaking from freezer stock onto tryptic soy agar (TSA) (VWR International Srl) and overnight incubation at 37 °C, overnight liquid cultures were then maintained in tryptic soy broth (TSB) at 37 °C and with continuous shaking at 200 rpm to prepare the inoculum for experiments. Appropriate positive controls (Vancomycin antibiotic) and negative controls (vehicle control and sterile media control) were incorporated into the assays.

### 2.6 Growth Inhibition Assays

The crude extracts (2702 and 2707), fractions, SFs, and punicalagin (α and β mixture) are of were examined by dose–response experiments to obtain the half-maximal inhibitory concentration (IC_50_) and minimum inhibitory concentration (MIC) values against LAC. Growth inhibition was determined by a change in optical density (OD) readings at 600 nm from the start of incubation to the final time point (18 h) relative to vehicle control (DMSO).

All growth inhibition experiments were carried out following the guidelines set by the Clinical Laboratory Standards Institute (CLSI) M100-S23, for broth microdilution testing.^21^ Briefly, standardized working cultures were calculated and diluted from TSB overnight cultures in cation-adjusted Müller-Hinton broth (CAMHB) to an OD_600_ of 0.0006, which corresponds to 5 × 10^5^ CFU/mL using a Cytation 3 multimode plate reader (Biotek). Using 2-fold serial dilution, extracts, and vehicle control at concentrations ranging from 8 to 256 μg/mL, and antibiotic (Vancomycin) ranging from 0.5 to 16 μg/mL were included in the plate setup, and the assays were performed in 96-well flat-bottom nontissue culture-treated plates (Falcon 35-3075, Corning, NY, USA). After treatment, plates were statically incubated at 37 °C for 18 h. OD_600_ nm was measured using a BioTek Cytation3 plate reader at initial and final time points, to account for extract color, and the percent inhibition was calculated as previously described.^22^ A media blank was included in each experiment to test for contamination; all concentrations were tested in triplicate, and experiments were performed at least twice on different days to account for two biological replicates to confirm the accuracy of the results. The MIC was determined as the lowest treatment concentration at which a 90% or greater reduction in optical density was achieved compared with vehicle control and the IC_50_ was defined as the lowest concentration tested at which at least 50% of growth was inhibited. Dose–response curves were generated using GraphPad Prism ver. 10.1.0 software.

### 2.7 Human Keratinocyte Toxicity Assay

*In vitro* dose–response cytotoxicity of active fractions to immortalized human keratinocytes (HaCaTs) was assessed following the lactate dehydrogenase (LDH) assay manufacturer’s instructions (LDH assay kit, G-Biosciences, St. Louis, MO) as previously described.^22^ Briefly, upon reaching suitable cells confluency (70–90%), HaCaT cells were standardized to 4 × 10^4^ cells/mL, and 200 μL of cell culture was added to wells in 96-well tissue culture microtiter plates and incubated for 24 h to allow for seeding. After incubation, treatments, and fresh media were added to HaCaT cells at a concentration range of 16–128 μg/mL via serial dilution. Plates were subsequently incubated at 37 °C with 5% CO_2_ for 24 h, and cells were then processed according to the manufacturer’s protocol for chemical-induced cytotoxicity. All tests were performed in triplicate, and the full experiment was repeated on a separate day using fresh cell stock.

### 2.8 Quorum Sensing Inhibition Assay

 fractions and crude extract were examined by a dose–response assay for quorum sensing inhibitory activity against the accessory gene regulator (agr I) strain AH1677 as previously described.^23^ The gene regulator strain was grown and maintained on TSA and then TSB, supplemented with chloramphenicol (10 μg/mL) at 37 °C while shaking at 200 rpm. The overnight AH1677 cultures were standardized to an OD_600_ value of 0.0006 for working cultures. Serial dilutions (4 to 128 μg/mL) were made of fractions and crude extract in 96-well black plates (Costar 3,603, final well volume: 200 μL). Plates were incubated in a humidified chamber at 37 °C while shaking at 1,200 rpm (Stuart SI505 incubator, Bibby Scientific, Burlington, NJ). Fluorescence (top reading, 493 nm excitation, 535 nm emission, gain 60) and OD_600_ nm readings were taken with a plate reader (BioTek Cytation3) at 0 and 18 h post-inoculation. Controls including a vehicle control (DMSO), and a positive control (224C–F2) were also assessed from 4 to 128 μg/mL. 224CF2c is a QSI-active fraction extracted from the European chestnut (), as reported in a previous study by the authors.^22^ All tests were performed in triplicate and repeated using a new stock of bacteria on two different days to achieve appropriate biological and technical replicates. Data was analyzed using Microsoft Excel, and figures were created with GraphPad Prism version 10.1.0.

### 2.9 In Food Matrix Bactericidal Activity toward Induced Contamination

Minced meat and cheese samples were obtained from a local grocer, and the absence of contamination with had been certified. The in-food matrix antibacterial activity of PPE against DSM 20231 and DSM 25691 strains was evaluated as previously described^24,25^ with some modifications. Additionally, the method of Martínez et al. (2019) was followed to avoid the contamination of fresh cheeses with that occurs during handling.^26^ Briefly, bacterial cultures were maintained overnight in TSB (VWR International Srl) broth to prepare a working solution. After incubation, the concentration of the bacterial suspension was centrifuged, and the cell pellet was washed twice with sterile distilled water and adjusted to a final concentration of 1 × 10^6^ cells/mL in a sterile buffered peptone water solution. Subsequently, cultures were inoculated individually in cheese and meat samples to simulate contamination before pomegranate peel extracts treatment.

Minced meat samples were then treated with 200 mg of lyophilized powder PPE of (2707) and were aseptically homogenized and individually packed in presterilized polyethylene bags and stored at 5 °C. Negative controls (inoculated with but not treated with the PPE) were also prepared and subsequently stored with treated samples in a laboratory refrigerator for periodic total bacterial count determination on days 0, 3, and 7 (three replicates for each time point for each strain). The same procedure was followed with cheese, where samples were soaked in 25 mL of PPE (2707) for 10 min at a concentration of 4.5 mg/mL. Next, samples were individually packed in presterilized polyethylene bags and stored at 5 °C. Negative controls (inoculated with but not treated with the PPE) were also prepared and subsequently stored with treated samples in a laboratory refrigerator for periodic total bacterial count determination on days 0, 7, 15, and 30 (two replicates for each time point for each strain).

Total viable counts of in stored samples were evaluated. On each analysis time point, appropriate serial dilutions ranging from 10^–1^ to 10^–3^ of the obtained homogenate were prepared. 100 μL of each dilution were plated onto Baird Parker agar (BP) (VWR International Srl), which is selective media for , and plates were then incubated at 37 °C for 24 h. Each microbiological count was performed in triplicate and is expressed as log_10_ CFU/mL.

### 2.10 Statistical Analyses

Data analysis was conducted using GraphPad Prism v.10.2.2. Pairwise comparisons for antimicrobial activity in food model assays and differences between groups at specific time points were assessed with a two-tailed, nonparametric Mann–Whitney unpaired rank-sum test.

## 3 Results

### 3.1 Bioassay-Guided Identification of Active Compounds from 

 fruit peel extractions were achieved by means of maceration in 80% ethanol yielding 7 crude extracts belonging to 7 different pomegranate varieties. Based on the chromatographic data and findings from the prior study,^14^ only the most potent varieties were chosen for further investigation. Bioassay-guided fractionation of these organic extracts (named extracts 2702 and 2707) was directed by a set of strains assays. The fractionation of the crude extracts for compound isolation was achieved by reversed-phase prep-HPLC using a gradient system of water and acetonitrile. A first round of prep-HPLC yielded 16 fractions 2702 (PF1, PF2, PF3, PF4, PF5, PF6, PF7, PF8) and 2707 (PF1, PF2, PF3, PF4, PF5, PF6, PF7, PF8). Subsequently, the most bioactive two fractions, 2707-PF5 and 2707-PF6, were selected for a second round of prep-HPLC subfractionation and chemical analysis because of their good antibacterial activity both in growth inhibition with MIC values of 64 μg/mL and because of their lack of toxicity toward human cells. The second round of preparative HPLC led to the generation of 68 SFs. Out of these 68 SFs, only a few SFs showed good antimicrobial activity with MIC between 32 and 64 μg/mL and underwent further structure determination using liquid chromatography with mass spectrometry (LC-MS). The compounds of the most active SFs were identified by mass spectrometry (MS). Putative matches were only obtained for peak numbers 1 and 2 with an empirical formula of C_48_H_28_O_30_, which corresponded to 5 compounds (α-punicalagin, β-punicalagin, isoterchebulin, terchebulin, punicacortein C) in the database Reaxys (Figure S1). Among them, 3 compounds (α-punicalagin, β-punicalagin, punicacortein C) were reported from the *Punica* genus. The identification of peaks 1 and 2 as two ellagitannins punicalagins α and β was performed by comparison of LC-MS data with a standard compound (punicalagins α and β mixture), *m*/*z* values of 541.0260 and 541.0255. The two anomer structures were determined by employing spectroscopic analyses and comparisons with literature data (Figure 1).

**Figure 1:**
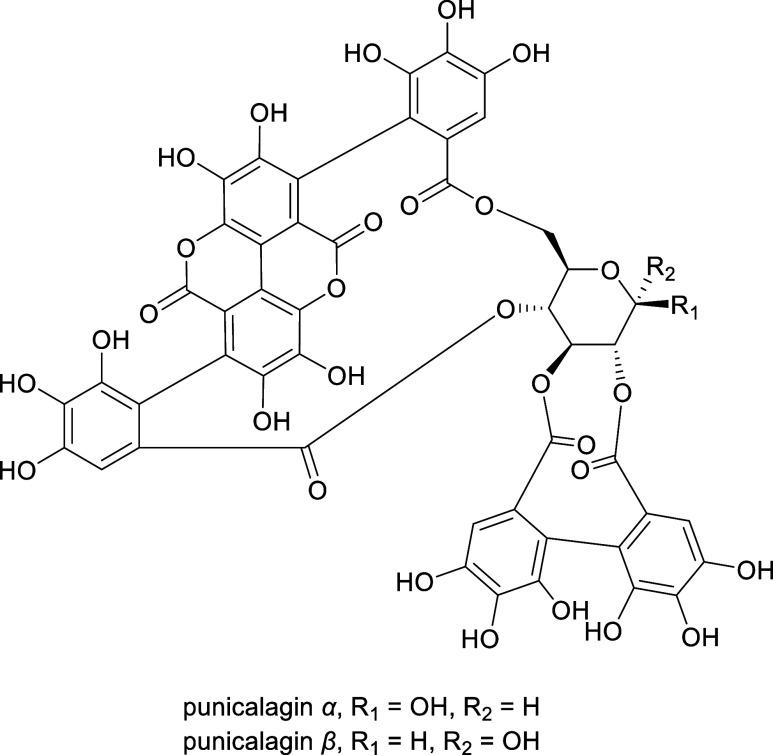
Chemical structure of punicalagin anomers α and β, α-punicalagin punicalagin α (CAS # 130518-17-1) and punicalagin β (CAS # 30608-10-5).^27^

### 3.2 Growth Inhibition

To determine their activity against strains, and a total of 87 samples of crude extracts (2702 and 2707), their fractions, SFs, and punicalagin (α and β mixture) were investigated for growth inhibition by dose–response experiments to obtain the IC_50_ and MIC values. Of the first antimicrobial screening for the crude extracts for both 2702 and 2707 and their fractions, a good number of the tested samples displayed potent antimicrobial activity against the tested LAC strain with MIC values ranging from 64 to >256 μg/mL and IC_50_ values ranging from 16 to 128 μg/mL (Figure 2).

**Figure 2:**
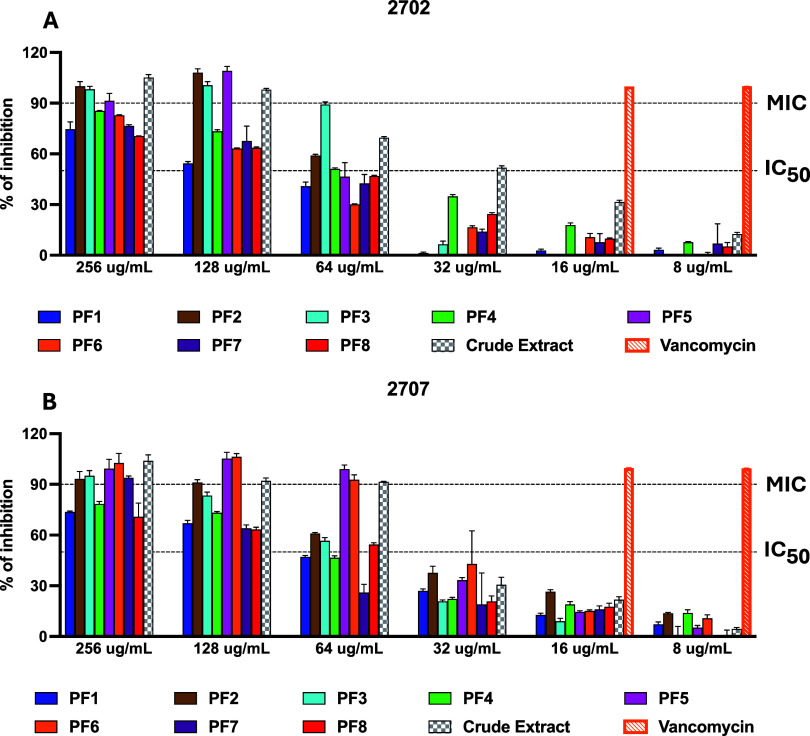
crude extracts (A) 2702 and its fractions and (B) 2707 and its fractions exert selective concentration-dependent inhibition of LAC at 18 h post-inoculation. Vancomycin was used as a positive control for the growth inhibition.

Based on the MIC observed values, only two fractions 2707-PF5 and 2707-PF6 were processed for further fractionation. This step yielded 68 SFs which were tested for their antimicrobial activity (Figure S2). The tested SFs showed good antimicrobial activity against the tested LAC strain, Figure 3 reports the minimum concentrations of the tested samples for both IC_50_ and MIC for the most active SPs ranging from 32 to 64 μg/mL. Finally, by testing the antimicrobial activity of Punicalagins α and β, the MIC value was 16 μg/mL and IC_50_ was 4 μg/mL.

**Figure 3:**
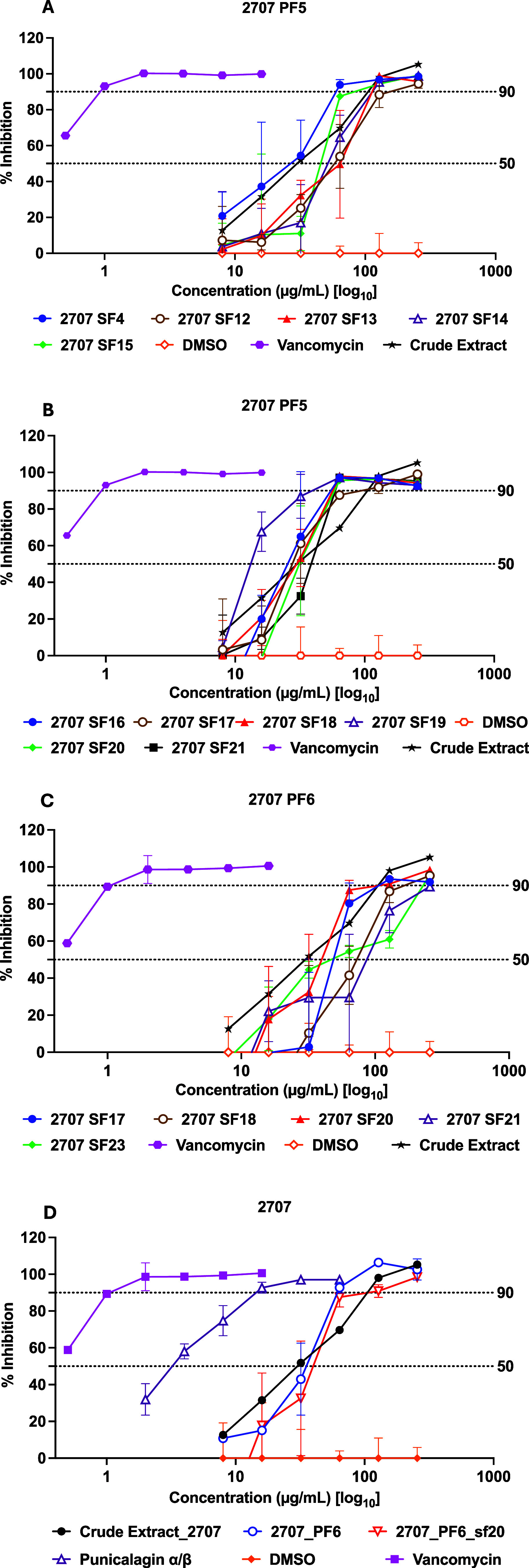
Selective concentration-dependent inhibition of LAC at 18 h post-inoculation by 2707 fractions: (A, B) PF5 subfractions, (C) PF6 subfractions. (D) Illustration of the complete fractionation process. Vancomycin was used as a positive control for growth inhibition.

### 3.3 Quorum Sensing Inhibition in 

The potential of crude extracts 2702 and 2707, along with their respective fractions, to inhibit quorum-sensing pathways regulated by the accessory gene regulator (agr) system was evaluated using an agr I fluorescent reporter strain (AH1677). None of the tested samples exhibited quorum-sensing inhibitory activity at concentrations up to 128 μg/mL. The slight reduction in agr I transcriptional activity was attributed to general growth inhibition of the reporter strain rather than specific QS interference (Figure S3). Although quorum sensing regulates virulence and biofilm formation in , punicalagin showed no antiquorum sensing activity in our assay, indicating that its antimicrobial effects likely operate through QS-independent mechanisms.

### 3.4 Cytotoxicity of Active Fractions

To determine the potential toxicity of fractions to human cells, HaCaT cells were tested in a dose–response study using a lactate dehydrogenase assay to assess their cytotoxicity. Of the 16 fractions studied, 3 fractions (2702 PF3, 2707 PF5, and 2707 PF6) were recognized to have potential antimicrobial activity and were tested for potential cytotoxicity. The fractions were tested at a starting concentration of 128 μg/mL. Figure 4 displays cytotoxicity across the tested samples, demonstrating that none of the fractions tested had high cytotoxicity with an IC_50_ greater than 128 μg/mL.

**Figure 4:**
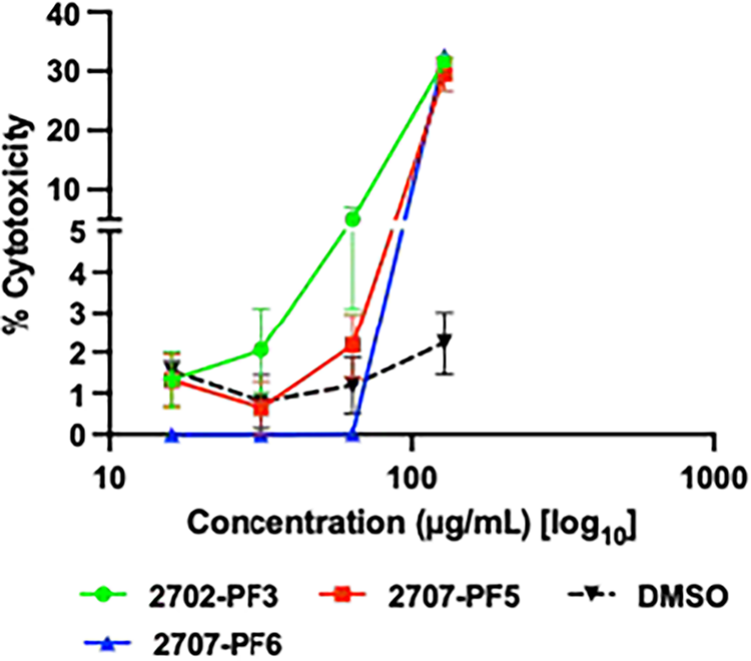
Human keratinocyte cytotoxicity by , the most active fractions. Figure made with GraphPad Prism version 10.1.0 for Windows, www.graphpad.com.

### 3.5 In Food Matrix Bactericidal Activity toward Induced Contamination

The impact of pomegranate crude extract 2707 on the shelf life of commercially available minced meat and cheese was investigated. A significant influence of PPE supplementation was identified, where it was observed that the addition of peel extracts preserved the quality of minced meat and cheese throughout the storage duration. A considerable reduction trend during the storage period in both control and treated samples was observed by the total plate count and the examined food models demonstrated a significant (*p* < 0.05) reduction in *Staphylococcus* strains ( DSM 20231, and DSM 25691) counts in treated samples when compared to the untreated control (Figures 5 and 6). Additionally, mold growth was observed after 10 days of incubation on untreated cheese plates but not on treated ones. For meat samples, color change was visually observed on day 3 for control samples and on day 5 for treated samples.

**Figure 5:**
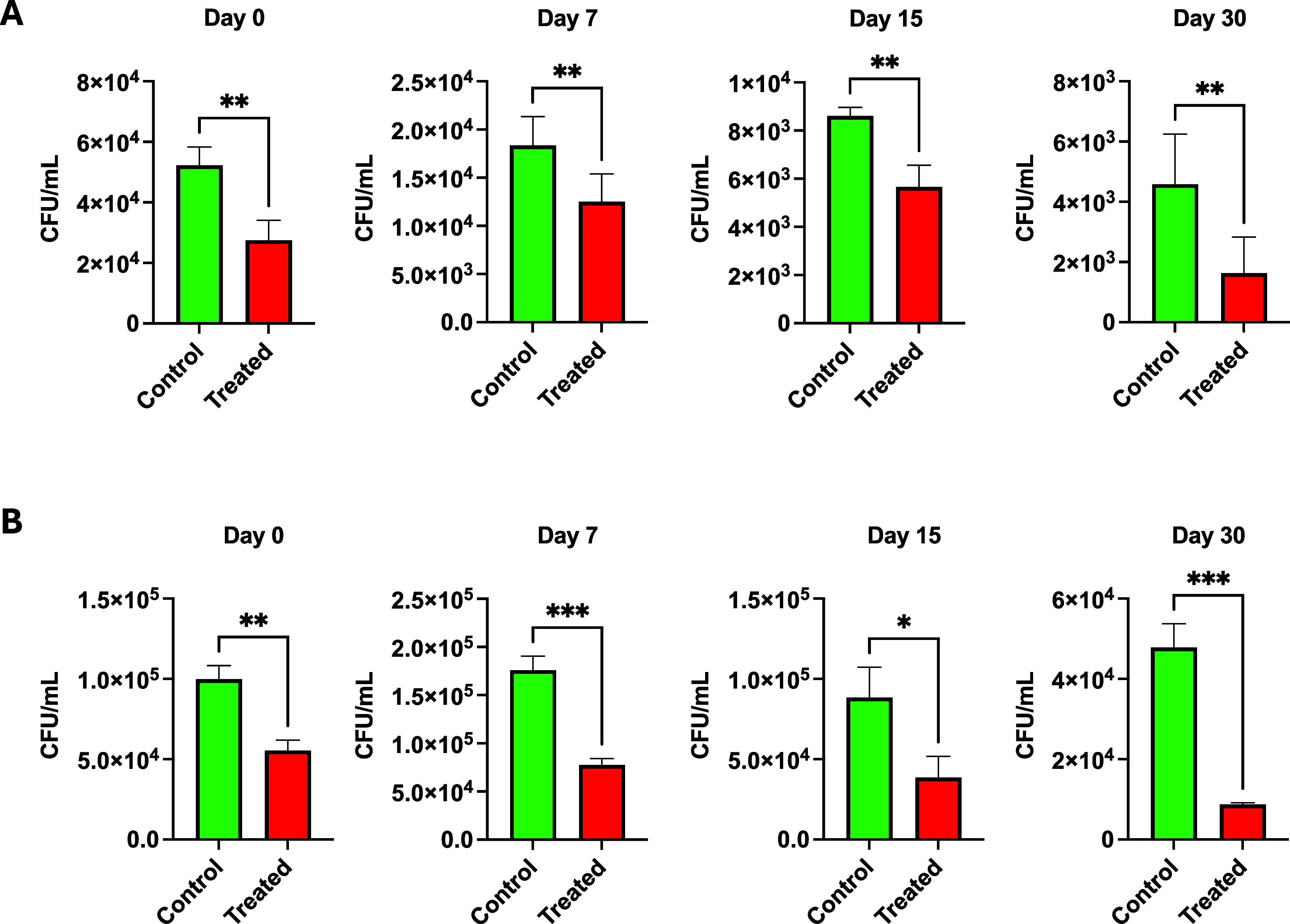
In-food matrix pomegranate peel extract (2707) bactericidal activity toward induced contamination using a cheese food model over 0, 7, 15, and 30 days. A: 20231 B: 25691. Asterisks denote statistically significant differences between the groups, as measured by Mann–Whitney rank-sum test (two-tailed, ***P < 0.001, **P < 0.01, and *P < 0.05).

**Figure 6:**
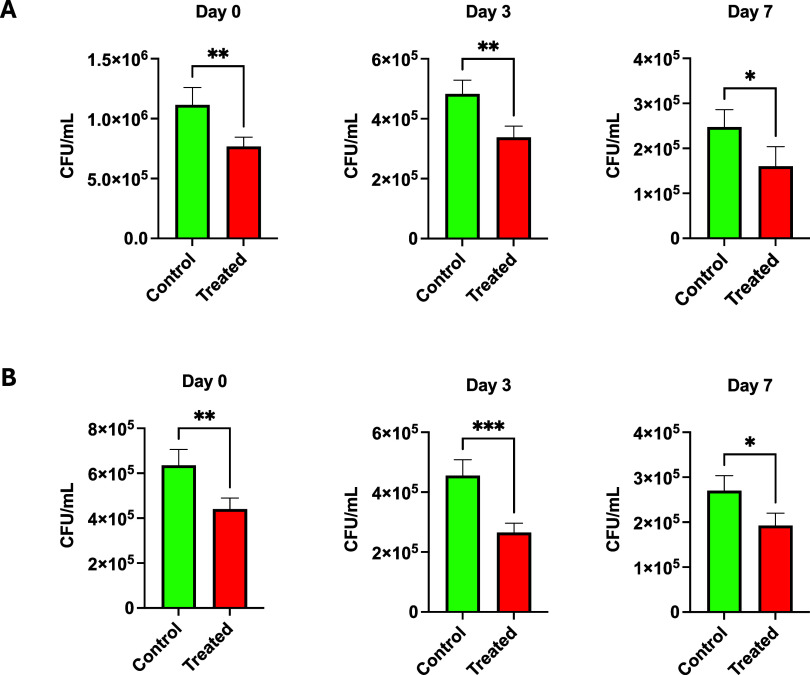
In-food matrix pomegranate peel extract (2707) bactericidal activity toward induced contamination using minced meet food model over 0, 3, and 7 days. **A**: 20231 **B**: 25691. Asterisks denote statistically significant differences between the groups, as measured by Mann–Whitney rank-sum test (two-tailed, ***P < 0.001, **P < 0.01, and *P < 0.05).

## 4 Discussion

### 4.1 Bioassay-Guided Isolation of Active Compounds and *In Vitro* Assays

Polyphenols found in pomegranates have been shown to exhibit diverse pharmacological and physiological effects, including but not limited to anticancer, antioxidant, antibacterial, and anti-inflammatory properties.^28–30^ Specifically, pomegranate peels contain substantial levels of hydrolyzed ellagitannins, including punicalins, punicalagins, and pedunculagins. Additionally, aside from ellagitannins, the peel of the pomegranate also contains hydroxybenzoic acids, such as gallic acid and ellagic acid, as well as anthocyanidins and flavonoids.^31,32^

As a part of the ongoing efforts to identify natural products as antibiotics, in the current study, two ellagitannins, Punicalagins α and β, were identified and isolated from peels. Punicalagin is recognized as the primary bioactive compound in pomegranates due to its abundance and biological activity.^33,34^ These compounds were also detected and isolated from the leaves, seeds, and juice of .^35,6,16^ The dose–response assay revealed the antimicrobial activity for identified Punicalagins α and β against LAC strain with an MIC of 16 μg/mL. The antibacterial activity of the punicalagins α and β anomeric forms, both *in vitro* and *in vivo* have been reported by several authors.^36,4,33^ Per our results, the antibacterial activity for punicalagin MIC was established as 61.5 μg/mL.^35^ Another study,^37^ reported that punicalagin exhibited an antistaphylococcal effect with an MIC of 250 μg/mL; however, it showed a moderate inhibitory effect on *Salmonella* with an MIC of 250–1000 μg/mL. Punicalagin demonstrated a significant antimicrobial impact and effectively inhibited the formation of biofilms by , suggesting potential applications for controlling contamination in the food industry.^37^

The antimicrobial effects of pomegranate are linked to polyphenolic tannins, particularly punicalagin and ellagic acid present in the extract, and involve various independent mechanisms.^17^ One proposed antimicrobial mechanism for polyphenolic compounds involves their ability to precipitate with proteins in the bacterial cell membrane, resulting in the lysis of bacterial cells.^38^ Moreover, polyphenols may hinder microbial enzymes by interacting with sulfhydryl groups or engaging in nonspecific interactions with proteins.^39^ Furthermore, it has been documented that polyphenols can impair the microbial respiratory chain by reducing oxygen consumption, thereby restricting the oxidation of NADH.^40^

Cooper et al. (2018) investigated the inhibitory effects of punicalagin on and found that it disrupts bacterial growth by impairing iron homeostasis and triggering the SOS response, likely through inhibition of DNA biosynthesis.^41^ Further analysis revealed that punicalagin treatment significantly altered the bacterial proteome—suppressing proteins and enzymes critical for iron uptake while simultaneously inducing an SOS response to DNA damage—indicating its potential to limit bacterial colonization by targeting key metabolic pathways.^42^ Moreover, punicalagin impaired SrtA-associated virulence traits *in vitro* by reducing adherence to fibrinogen, decreasing the surface expression of protein A (SpA), and inhibiting biofilm formation. These effects were supported by fluorescence quenching analysis, and molecular docking studies showed that punicalagin binds to key SrtA residues, including LYS190, TYR187, ALA104, and GLU106.^4^ These findings will prove valuable to researchers in the fields of antibiotics and *Staphylococcus*, aiming to safeguard public health and enhance food safety for example punicalagin, with its notable natural properties, could serve as an effective additive for meat preservation and quality improvement, potentially offering a viable alternative to synthetic antioxidants.^6,43^

Quorum-sensing (QS) inhibitory activity was also evaluated for the pomegranate peel extracts in this study. The results revealed that none of the investigated samples demonstrated QS inhibitory effects against the agr system. This contrasts with several previous studies reporting the QS-modulating potential of extracts. For example, Ismaeil and Salih (2020) reported that various pomegranate extracts inhibit QS in , downregulating key QS and virulence-related genes such as *sea*, *seb*, *agrA*, *RNAIII*, and *hla*.^44^ Similarly, punicalagin, a major pomegranate polyphenol, was found to suppress virulence factor expression and QS signals in *Salmonella* at subinhibitory concentrations.^37^ Hamrita et al. (2022) also observed anti-QS activity of methanolic pomegranate extract against , including significant inhibition of swarming motility and reduced pyocyanin production at low concentrations.^45^ A comparative analysis found that pomegranate peel extract had a weaker effect on violacein production in than several other medicinal plants, suggesting that its quorum-sensing inhibition capacity may be limited or context-dependent.^18^

The difference between our results and those from previous studies may be due to variations in pomegranate types, extraction methods, and the tested bacteria. For instance, a study by Abutayeh et al. (2024) demonstrated that the antibacterial efficacy of PPEs varied significantly based on the extraction technique and solvent employed. Specifically, aqueous macerate and microwave-assisted extracts exhibited high potency against , , and . In contrast, showed greater susceptibility to ethanolic extracts, highlighting the role of solvent polarity in antimicrobial activity.^46^ Although the pomegranate peel extract showed strong antimicrobial activity against , it did not inhibit quorum sensing under our conditions. This suggests that the extract acts through direct antibacterial effects rather than disrupting bacterial communication. Future studies should use standardized QS tests, include more bacterial strains, and explore how different doses affect the results. Advanced tools, such as gene expression analysis, could further explain how the extract interacts with QS pathways.

In cytotoxicity assays with human keratinocytes, the pomegranate fractions showed selectivity for bacterial cells over mammalian cells, where none of the fractions tested had high cytotoxicity with IC_50_ greater than 128 μg/mL. Such selectivity was also reported by Kilit and Aydemir (2023) where they observed that punicalagin showed cytotoxicity against several cancer cells but was not cytotoxic against human kidney epithelial cells.^47^

### 4.2 *In Vivo* Bactericidal Activity toward Induced Contamination

The widespread resistance of numerous microorganisms to existing antibiotics is a major concern worldwide. This issue, coupled with the increasing consumer focus on “natural food products,” has motivated researchers and the food industry to explore novel alternative compounds capable of effectively inhibiting a wide range of microorganisms.^48^ As a result, the popularity of employing plant extracts as natural antimicrobial agents for food preservation is on the rise.^24^ In the present study, aqueous PPE (2707) was used as a natural antimicrobial agent in food preservation. A significant reduction of up to 0.8-fold in bacterial cell counts was observed for minced meat and cheese after the incorporation of PPE into the tested food models. This aligns with the findings of several previous studies where it was observed that incorporating PPE led to a remarkable reduction in the total bacterial plate count, contributing to the preservation of meat products’ freshness during refrigerated storage and had a positive effect on color stabilization.^49,50^ A study was conducted by Parafati et al. (2021) to investigate the antimicrobial potential of pomegranate extracts against when integrated into the cheese matrix, cheeses showed a decrease in counts, of more than one log unit in comparison to the control cheese.^25^ In addition, Mahajan and Kumar (2015) observed a significant effect on the microbiological characteristics of cheese when treated with PPE against the different tested strains of bacteria, yeast, and mold where lower count values were recorded when compared to control.^51^ Employing an alternative food model, it was observed that untreated control samples of chicken spoiled within a week storage period, whereas treated samples exhibited an extension in shelf life, lasting up to 20 days.^24^

Also in the present study, mold growth became apparent after 10 days of incubation in the refrigerated untreated cheese samples, while no such growth was observed in the treated samples. This can be attributed to the antifungal properties of PPE, where many studies have already documented the potential antifungal properties of extracts from pomegranate peels and seeds, indicating their potential as natural substitutes for antifungals.^52,53^ Additionally, in a study where Nawaz et al. (2025) evaluated the antifungal activity of PPE against nine pathogenic fungi, including , , and . The *n*-hexane fraction of the ethanolic extract exhibited the highest inhibition efficacy. Notably, the polyphenol compound nobiletin demonstrated strong inhibitory effects against , , and .^54^ Although this study focused on antibacterial activity, future work should include antifungal assays to further evaluate the compounds’ antimicrobial potential.

Hence, incorporating pomegranate byproducts containing notable bioactive properties not only improves quality and prolongs shelf life by inhibiting oxidative damage to proteins and lipids but also enhances the functional and health-related characteristics of products such as meat, fish, milk, and their derivatives during storage.^55^

In this study, a concentration of 4.5 mg/mL was employed, exceeding the recorded value of the MIC for pomegranate extract (2707) that was reported before this study (0.75 mg/mL),^14^ to observe notable inhibition of bacterial growth. The differences between the *in vitro* and *ex vivo* values could be attributed to many reasons, where Smith-Palmer et al. (2001) determined that the antimicrobial efficacy of specific natural compounds was markedly impacted by the chemical composition of cheese.^56^

A high content of proteins in meat products such as ground beef and poultry can diminish the antimicrobial efficacy of plant-based compounds, as has been widely documented.^57^ Similarly, milk fat has been shown to interfere with the activity of essential oils and phenolic compounds. For instance, Alves et al. (2016) reported that the combined antimicrobial effects of carvacrol, thymol, and eugenol against were significantly reduced in cow milk compared to a standard culture medium.^58^

Similarly, Gutierrez et al. (2008) observed a decline in the antimicrobial potency of oregano and thyme essential oils against when exposed to increased lipid levels in a simulated food matrix.^59^ Hence, to attain an inhibitory effect comparable to the one observed *in vitro*, it is necessary to incorporate the same natural compounds in foods at higher concentrations.^60^ This was also confirmed by Gammariello et al. (2008) where they observed that achieving a similar antimicrobial effect in cheese necessitated higher concentrations of the examined natural compounds compared to those used *in vitro*.^61^ Additionally, in a study to improve ground beef preservation using carvacrol-loaded polylactic acid films, it was recorded that partitioning of carvacrol into the fat phase of the beef reduced its antimicrobial activity.^62^ In conclusion, this study highlights the potential of pomegranate peel extract (PPE) and its bioactive component, punicalagin, as promising natural antimicrobial agents against . Using a bioassay-guided fractionation approach, we successfully isolated and identified compounds with potent antimicrobial activity, particularly punicalagin, which was shown to disrupt iron homeostasis and attenuate virulence traits in . These findings suggest applications beyond food preservation, including the development of antimicrobial coatings for medical devices and surfaces as well as pharmaceutical formulations for topical treatment of skin infections and wound healing therapies targeting antibiotic-resistant pathogens. Furthermore, the evaluation of safety profiles supports the feasibility of PPE-based products as safer alternatives to synthetic antibiotics and preservatives. This work contributes to the growing body of research on repurposing agricultural byproducts, positioning PPE as a novel, cost-effective, and eco-friendly solution for combating antimicrobial resistance across multiple sectors, including healthcare, pharmaceuticals, and food industries.

## Supplementary Material



## Notes

LC-MS data of punicalagins α (peak1) and β (peak2) in fraction 2707-PF6-sf20 and standard compound mixture (**SI** Figure 1); bioassay-guided fractionation of crude extract (2707) showing percent yield and growth inhibition MIC against AH845.Y (**SI** Figure 2); 2707 crude extract and its fractions showing negative quorum sensing inhibition of agr system at 18 h post-inoculation (**SI** Figure 3) (PDF)
