# Supplementary material for: Identification of Punicalagin as a Key Bioactive Compound Responsible for the Antimicrobial Properties of L. Peel Extract against
Source: J Agric Food Chem. 2025 Jul 9;73(29):18280–90. doi: 10.1021/acs.jafc.5c02320 (PMC12291446; doi:10.1021/acs.jafc.5c02320)
Supplement: Supplementary file 1 [file jf5c02320_si_001.pdf]

Supporting information

Identification of punicalagin as a key bioactive compound responsible for the antimicrobial properties of *Punica granatum* L. peel extract against *Staphylococcus aureus*

Amira Salim[a], Lewis Marquez[b], Kishor Jakkala[c], Sunmin Woo[d], Marco Caputo[d], Francesco Fancello[a], Pierfrancesco Deiana[a], Mario Santona[a] Maria Giovanna Molinu[e], Cassandra L. Quave[d f*], Severino Zara[a*]

[a] Department of Agricultural Sciences, University of Sassari, Sassari 07100, Italy

[b] Molecular and Systems Pharmacology Program, Laney Graduate School, Emory University, Atlanta, GA 30322, USA

[c] Department of Microbiology and Immunology, Emory School of Medicine, Emory University, Atlanta, GA 30329, USA

[d] Center for the Study of Human Health, Emory University, Atlanta, Georgia 30322, USA

[e] National Council of Research, Institute of Food Production Sciences, Sassari 07100, Italy

[f] Department of Dermatology, School of Medicine, Emory University, Atlanta, Georgia 30322, USA

*Email: cassandra.leah.quave@emory.edu
Phone: +14047277065
*Email: szara@uniss.it
Phone: 079 229386

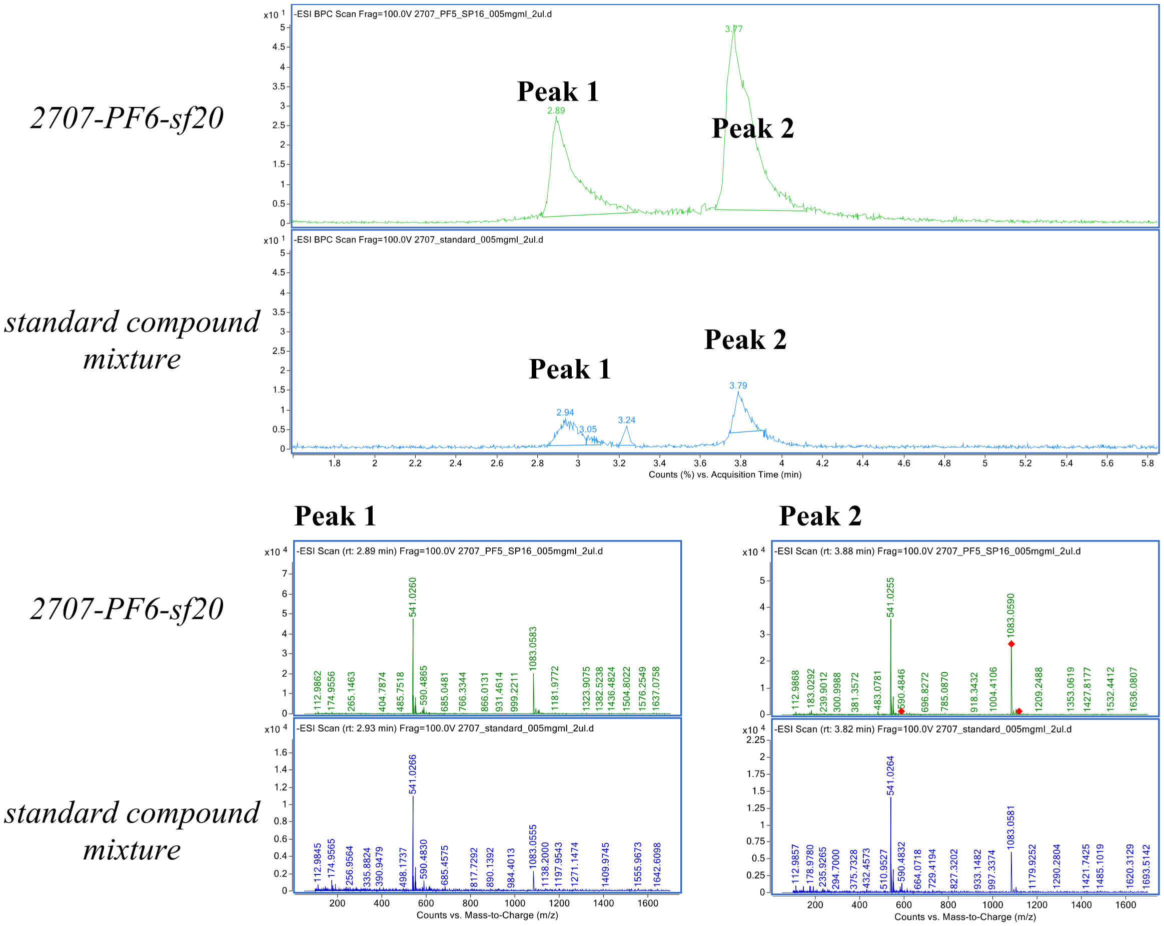

Figure S1. *LC-MS data of punicalagins α* (peak1) *and β* (peak2) *in fraction 2707-PF6-sf20 and standard compound mixture.*

Growth Inhibition MIC Hits against *S. aureus LAC*

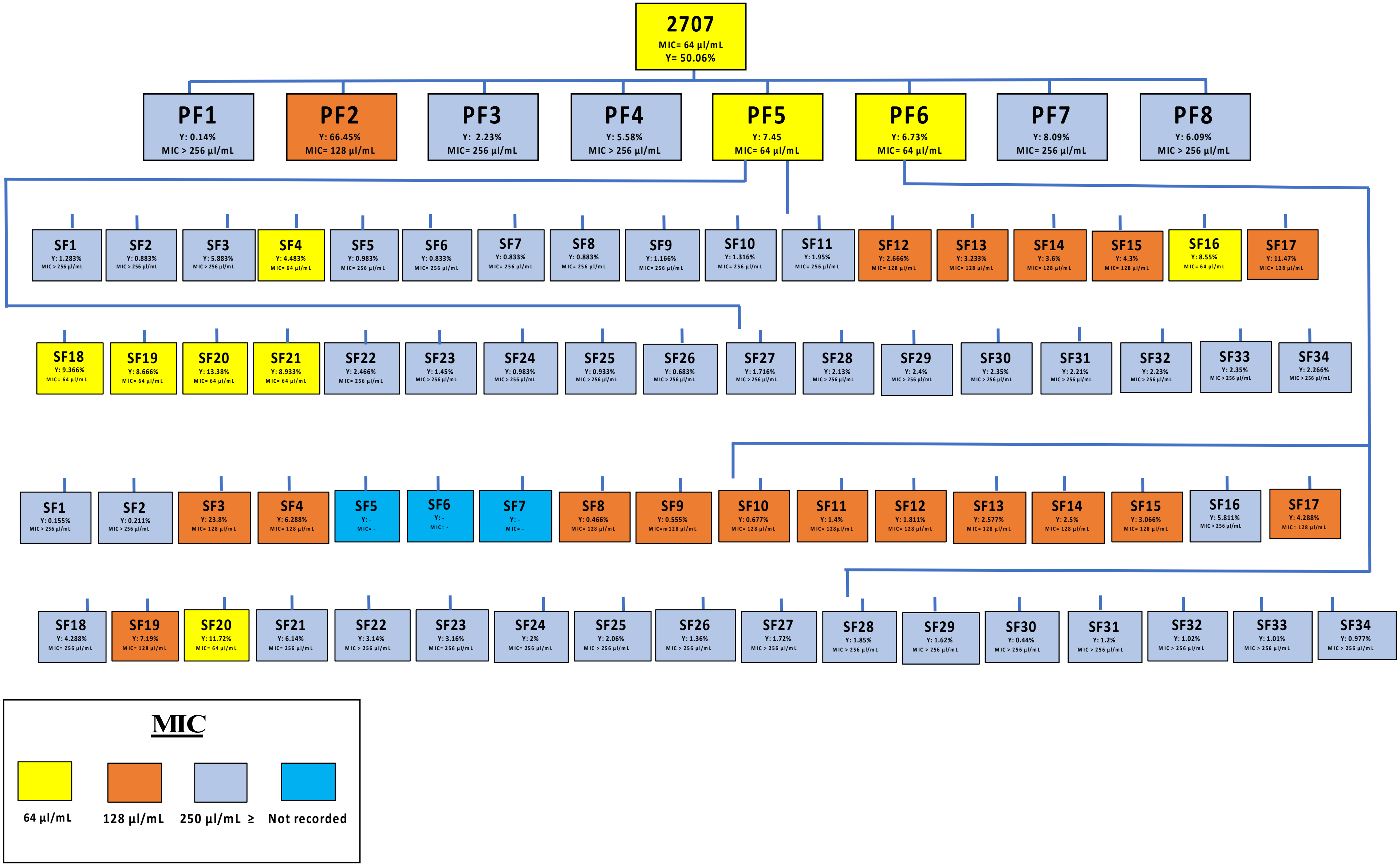

Figure S2. *Bioassay-guided fractionation of P. granatum crude extract (2707) showing percent yield and growth inhibition MIC against S. aureus AH845.Y: The yield of each fraction, where it is calculated relative to the parent; the yield of a crude extract is relative to dry plant material*

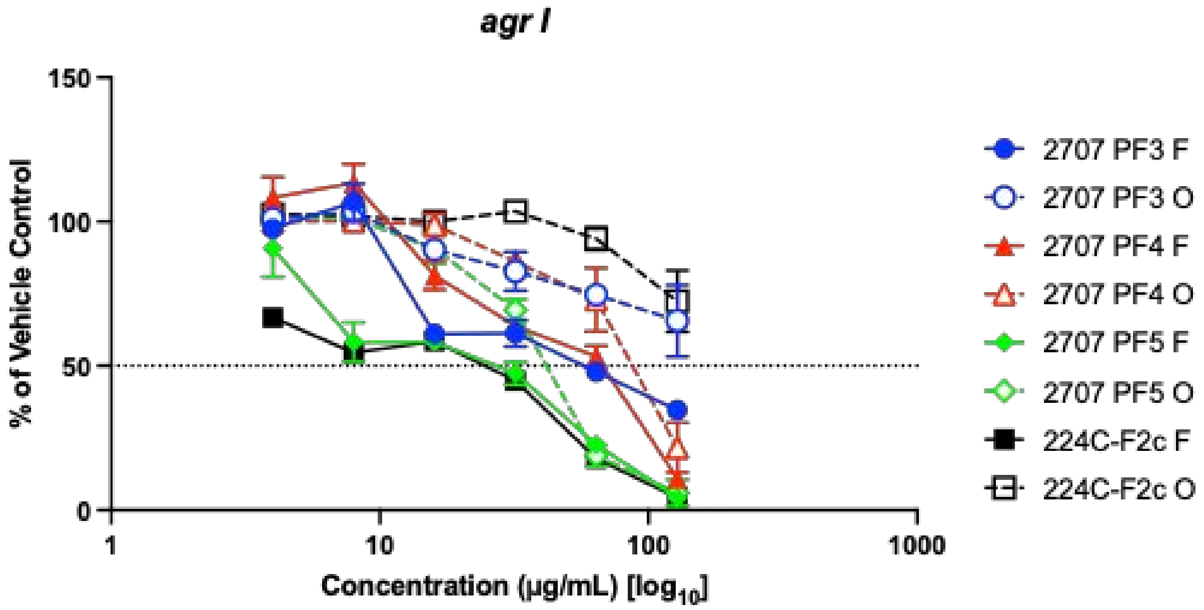
agr I
% of Vehicle Control
150
100
50
0
1
10
100
1000
Concentration (μg/mL) [log10]
2707 PF3 F
2707 PF3 O
2707 PF4 F
2707 PF4 O
2707 PF5 F
2707 PF5 O
224C-F2c F
224C-F2c O

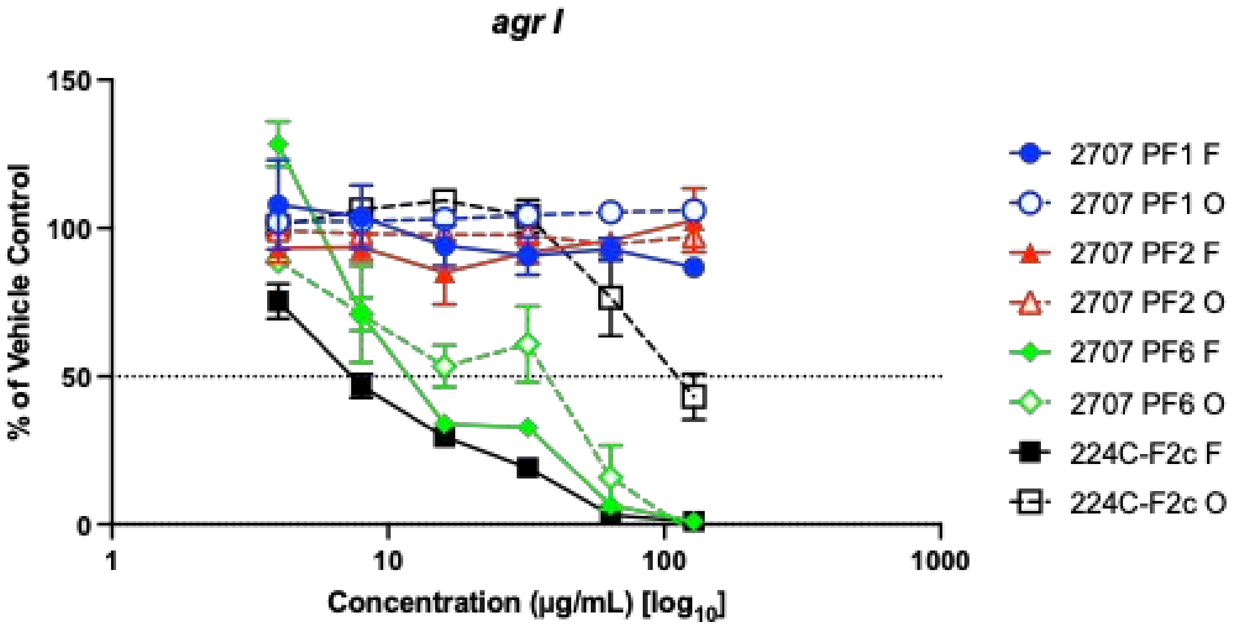
agr I
% of Vehicle Control
150
100
50
0
1
10
100
1000
Concentration (μg/mL) [log10]
2707 PF1 F
2707 PF1 O
2707 PF2 F
2707 PF2 O
2707 PF6 F
2707 PF6 O
224C-F2c F
224C-F2c O

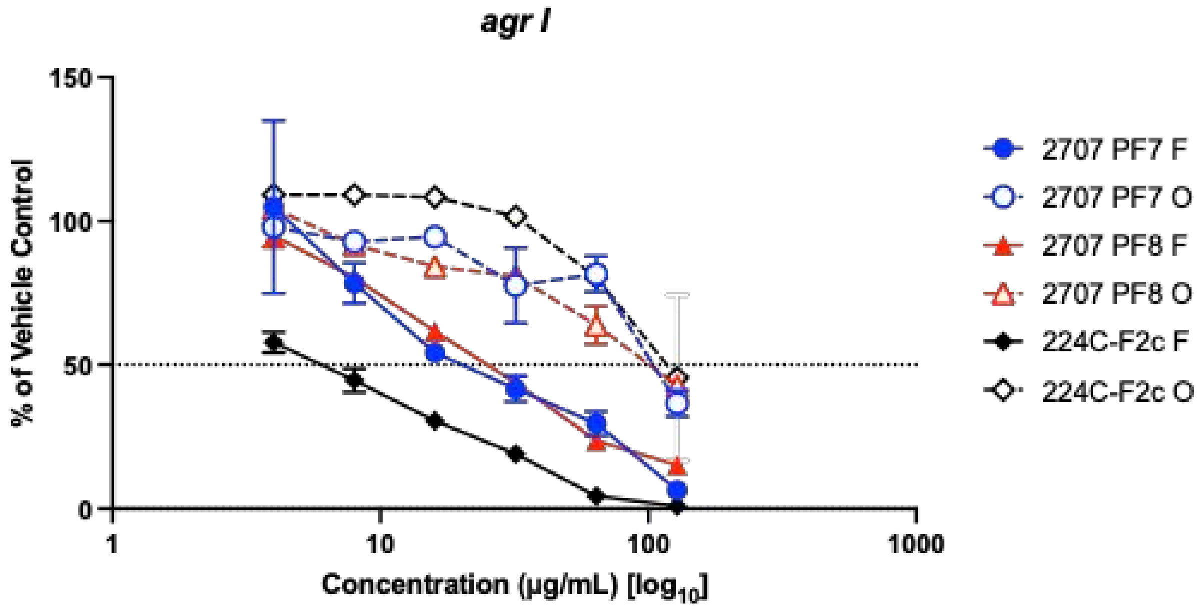

Figure S3. *P. granatum 2707 and its fractions showing negative quorum* sensing inhibition of *S. aureus agr system* at 18 hours post-inoculation. Vancomycin was used as a positive control for growth inhibition. All dashed lines represent optical density (OD) values, indicating growth; all solid lines represent fluorescence detection (FLD) values.
